# Breaking the taboo: qualitative analysis of the sexuality in people with acquired motor disability

**DOI:** 10.1186/s40359-023-01423-9

**Published:** 2023-11-08

**Authors:** Gema Sologuren-García, Carmen L. Linares, Jackeline R. Flores, Katty Mendoza-Mamani, Rinna M. Pilco, Gloria Escobar-Bermejo, Soledad Sotelo-Gonzales, Guicela Palza-Portugal

**Affiliations:** Jorge Basadre Grohmann National University, Tacna, Peru

**Keywords:** Motor disability, Experience, Sexuality, Discrimination

## Abstract

**Background:**

Globally 1 in 7 people experience some type of disability. In Latin America, as in other regions, there are programs focused on the protection of sexual and reproductive rights of this population group. However, to date, in Peru there are no programs, protocols or guidelines that include a specialist (sexologist or trained health professional) in the health system to improve the quality of life and well-being of this population. Therefore, the objective of this article is to qualitatively analyze the experience of sexuality in people with acquired motor disabilities.

**Methods:**

We used the interpretative phenomenological approach and the semi-structured in-depth interview composed of 60 questions, which was applied to 7 people (4 women and 3 men) with acquired motor disabilities.

**Results:**

Diversity in the experience of sexuality was observed. While some experience it without fear, others have annulled their sexual encounters, because in addition to physical limitations, they experience psychosocial limitations such as pain, functional alterations, depression, low self-esteem, discrimination, exclusion, stigmas and socio-environmental barriers, among others. Likewise, the study reflected the double discrimination suffered by women (for being a woman and having a disability), as well as the lack of education, counseling or sex therapy by specialized professionals, and the influence of religion, society and culture as factors that condition and limit their sexuality. This reflects the current situation in Peru and other countries in the region, where the sexuality of people with disabilities continues to be a complex and ambiguous issue.

**Conclusions:**

It is concluded that for some participants it is possible to experience sexuality without fear, while others hold back their experiences for reasons other than physical, self-esteem, social discrimination, among others. The review of the profile and level of specialization of the health services professionals involved in the integral medical care of people with disabilities and their couples is required because of their need for adequate and specialized attention for their better adaptation to the new condition.

## Introduction

Motor or physical disability is an acquired or born alteration, which compromises the body’s natural movement. It is caused by many conditions or disorders, which determine the degree and restrictions that the person has when performing activities in different spaces [1–4] This condition is present in the population worldwide. In 2011, the World Health Organization (WHO) indicated or that there are millions of people with some temporary or permanent disability, with less access to health care services, so that in many occasions their care needs are unattended [2]. Moreover, the inclusion of women with disabilities on the international agenda was first mentioned at the Second World Conference on Women in Copenhagen in 1980, in a brief declaration calling on governments to pay special attention to their needs, placing them alongside other vulnerable groups [5].

A report by the United Nations (UN), in 2019, recognizes that disability places women in a disadvantaged position, being excluded because of their gender role and disability; making women more vulnerable to the risk of violence, neglect and sexual abuse [5]Not being able to fully exercise their sexuality and have access to a full life of enjoyment is much more disabling than the disability itself [6]. In terms of sexual and reproductive rights, women with disabilities are more vulnerable than men because health care providers have little or no knowledge of the specific needs of their disability [7].

Worldwide, 1 in 7 people experience some type of disability, with an implicit assumption that each type has specific social, educational, and health needs [8]. In the Observatory of Physical Disability (ODF) of Spain, according to data from 2015, it is noted that of 1,505,645 women with physical-motor disabilities, 6% present high sexual satisfaction and 72% of them have low sexual satisfaction [9].

In Peru, according to the report of the last census conducted by the National Institute of Statistics and Informatics (INEI), 3 million 209 thousand 261 people of the total population in 2017; that is, 1 in 10 people, reported some type of disability. Of them, 1 million 820 thousand 304 people are women. The types of disabilities with the highest frequency are: Vision impairment (1 million 550 thousand 196 people), motor difficulties (to move or walk/use arms and legs) affecting 485 thousand 211 people and hearing difficulties affecting 243 thousand 486 people [10, 11].

The department of Tacna, headquarters of the research, is located in the southern region of the Peruvian territory on the border with Chile and Bolivia. It had, according to the last census of 2017, 286 thousand 240 inhabitants, of which 38 thousand 007 people, presented some type of disability, being the proportion 1 in 9 people, of which 21 thousand 904 are women. The most frequent types of disability are: visual impairment (18 thousand 165 people), people with two or more types of disability (7 thousand 529 people) and motor difficulties (5 thousand 704 people) [12]. These data ilustrates the vulnerable situation of this group, in relation to their human and sexual rights, especially that of women.

In Latin America, as in developed countries, there are programs focused on protecting the sexual and reproductive rights of people with disabilities [6, 13, 14]. In Peru, one of the agencies in charge of disability management is the National Council for the Integration of Poeple with Disabilities (CONADIS), created in 1998. Since that date, many advances and policies have been made for the attention of this group of people, which can be summarized in the creation of the Ministry of Women and Vulnerable People and legislation that promote social inclusion, rights, quality of life, accessibility to services, transportation, studies and others, related to different areas, ignoring and forgetting those concerning the affective-sexual area [15–17].

Despite the advances, to date there are no programs, protocols or guidelines that include a specialist (sexologist or trained health professional) in the Peruvian health system to improve the quality of life and well-being of the population with disabilities, as it exists in other countries, such as the Netherlands and Denmark, which include sexual assistance in their health programs as a right [18]. There is also no evidence of statistical data or research at the national or local level that addresses the needs of sexuality care for people with disabilities. Therefore, the objective of this research is to qualitatively analyze the experience of sexuality in people with acquired motor disabilities.

This research is relevant from a theoretical point of view, because it responds to the scarcity of information and studies on the sexuality of people with motor disabilities, especially in women. At a practical level, this study can improve the approach of health care professionals in this area. At a social level, the results can be used to support public policies that promote the recognition of the right to the full exercise of sexuality. In the academic context, it is relevant due to its originality and great implication because research shows that the subject is complex and there is still a lack of knowledge about the different possibilities of experiencing sexuality that people with disabilities have [19–23]. This research will expand the knowledge, doing justice to the perspective of these people.

## Method

Interpretative phenomenological analysis (IPA) was used as a qualitative research approach because it sought to understand the unique and special meanings that people with acquired motor disabilities give to the experience of their sexuality. These meanings constituted the raw material for this study. Therefore, IPA made it possible to explore, describe, interpret, understand and discover the common patterns to such experiences [24].

In line with the design, an in-depth interview was used, with a set of open-ended questions that allowed the researchers to obtain detailed information from the participants about their experiences [25], to understand the experience of sexuality in people with acquired motor disabilities. The interviews were conducted from March to May 2022 and lasted approximately 2 h each.

The interview guide was adapted from the questionnaire of Gaitán and Quevedo [26], based on the purpose of the research and the available literature. Following Rubio’s theoretical framework [27] used by Gaitán and Quevedo [26], the main questions were distributed into 4 categories or holons: gender, emotional bonding, eroticism and reproductivity (See Table 1). The final version of the interview guide contained 60 questions, which were reviewed by an expert in sexology. Then, the interview guide was tested for consistency and clarity. The results of this review process ensured and demonstrated the quality of the instrument.

**Table 1:** Structured results

CATEGORIES	SUB-CATEGORIES
Genre	Conception of being a man/woman Roles, stereotypes Discrimination
Affective bonding	Interpersonal relationships Heartbreak or Love Breakup
Eroticism	Privacy Meaning of Sexuality Sexual satisfaction Masturbation Pornography Forms of expression of the amatory Erogenous zones Sexual pleasure Difficulties in sexual encounters Sex education Sources of information Sexual training of the health care team*.
Reproductivity	Maternity and paternity Reproductive decision *

*Note*: The asterisk symbol (*) represents the emerging subcategories, which refer to subcategories that emerged during the organization of the information found in the interviews

### Participants and selection criteria

Snowball sampling was used to recruit people with motor disabilities in the city of Tacna. Eligible participants were contacted through the Regional Council for the Integration of People with Disabilities Tacna (COREDIS). In Peru, the National Council for the Integration of People with Disabilities (CONADIS) and a decentralized manner at the Regional level, the Regional Council for the Integration of People with Disabilities (COREDIS), are entities that manage the Registry of People with Disabilities, through a personal database of those who have some type of disability (number of people with these conditions, most frequent types, degree of severity, duration of this condition). For registration, people are evaluated according to the Technical Health Standard for the evaluation, qualification and certification of the person with disability of the Ministry of Health, approved with Ministerial Resolution No. 981–2016/MINSA. Each person has a Disability card, which is known as the “*Carnet de CONADIS*”, a document that is delivered by means of a resolution, where the type of disability is specified [28].

Inclusion criteria were: 18 years of age or older, with acquired motor disability, and with explicitly given informed consent. People with intellectual or sensory disabilities were excluded to ensure homogeneity of the sample, considering possible differences in the sexual experiences of people with other disabilities. The sample size was determined following the recommendations of Smith and Nizza [24] who suggest a sample size between 3 and 6 participants for studies using IPA as a research design. To ensure deeper meanings and higher data quality, 7 participants were included in the study, aged 35 to 65 years (See Table 2). Likewise, the data saturation criterion was applied. All participants reported a heterosexual orientation.

**Table 2:** Characteristics of the population under study

N°	Pseudonym	Sex	Age	Type of motor disability	Spinal cord injury
1	Evelyn	F	35	Physical motor disability (walking)	No
2	Gladys	F	47	Physical motor disability due to sequelae of poliomyelitis with hip dysplasia.	No
3	Small	F	57	Quadriplegic from paralytic poliomyelitis sequelae	No
4	Lila	F	57	Quadriplegic due to spinal cord injury at C5-C6 level with bladder and fecal incontinence.	Yes
5	Vita	M	45	Paraplegic due to spinal cord injury L1-L2	Yes
6	Josecito	M	49	For amputation of right lower limb (transfemoral).	No
7	Carlitos	M	65	Paraplegic, due to spinal cord injury, at L9, L10, with bladder and fecal incontinence.	Yes

### Data collection

Ethical approval for the study was obtained from the Institutional Research Ethics Committee of the Hipólito Unanue Hospital of Tacna-Peru, assigning it the code: CIÉI-HHUT: 10-CIÉI-2022. The purpose and procedures of the study were explained to the participants, who voluntarily signed their consent before the interview. Since confidentiality and anonymity were an important part of the research, pseudonyms were used, and were proposed by the participants.

The first author organized the interview schedule, which was individual. The interview guide was composed of open-ended questions, providing details to clarify them. Every interview was audio-recorded, lasting approximately 2 h each, and field notes were taken before, during and after the interviews. In order to conduct the interview, participants were moved to a specific location, as there were some access difficulties that prevented them from moving and making initial contact. Those adjustments were made to protect and guarantee physical and psychological their integrity. Participants did not receive incentives of any kind. The researchers noted that there was no conflict of interest.

### Data analysis

The audio-recorded interviews were transcribed word for word by the research team. The documents were then checked for accuracy by a person not associated with the study.

The first, second, and third authors independently reviewed the data, formed a coding framework through discussions, and then extracted categories and subcategories for analysis at research team meetings. Many meetings were convened to discuss, define, and revise themes until consensus was established.

To analyze and code the data, the interviews were transcribed anonymously, each one in a separate document. Then, the authors familiarized themselves with the data by reading the transcripts. At this stage, notes were taken in the margins, when necessary. This was followed by open coding and segmentation, stage where the researchers met permanently during the process to verify and contrast their preliminary findings. A book of codes and categories that integrated Rubio’s theoretical proposal was then elaborated [29] and merged with the inductive findings of the researchers. These categories grouped codes with similar contents, all linked to the exercise of sexuality (See Table 1).

## Results

Assuming Rubio’s theoretical proposal [27] called “holons of sexuality”, the findings were organized into four integrated categories, determined by biological, social and psychological factors. These work under a systemic model: (i) Gender holon, understood as the series of ideas, attitudes, values and concepts about what is understood by being a man or a woman, and the expectations that arise for each one of them [27]; therefore, it influences the formation of gender roles and stereotypes [30]. (ii) Affective bonding holon, addresses the interpersonal relationships that are established with others, recognizing within them positive feelings such as love, friendship and affection or negative feelings such as anger, resentment, and pain. Therefore, feelings are considered to be indispensable in the construction, maintenance and avoidance of bonds and relationships [29]. (iii) Eroticism holon, referring to the series of ideas, concepts and values that are created regarding sexual responses. Therefore, it is a human capacity to experience sexual pleasure, desire, and arousal, through biological and psychological stimulations that contribute to the construction of social representations and meanings. It should be clarified that this pleasurable response can occur at a personal level or with another person [30]. And, finally, (iv) Reproductivity holon, which refers to the human potentiality for reproduction, and also to the functions of maternity and paternity that could be exercised. At the psychosocial level, it involves decision-making and the autonomy to decide when, with whom and how to have children or not; as well as the capacity to care for others who are not exclusively their first-born.

In reference to the *gender holon*, it is evident that in the participants’ conception of being a man or a woman, there are no significant differences, except for sex. The interviewees refer that men are associated with traits of strength, protection, responsibility for the family, objectivity, reduced expressiveness, with greater freedom to occupy managerial positions; and they are not socially judged as women are. As for women, the interviewees associate them with procreation, perseverance, sensitivity, strength, intuition, caretaker of the harmony of the home, subjective, expressive, fighter, passionate and at the same time, more socially judged. At the same time, they point out that women suffer double discrimination (for being a woman and having a disability). The following testimonies illustrate the gender differences described by the participants:

*“To be a man is to have the strength to support your whole family, the strength to work, to teach your children to be good, to succeed, that is, to support your whole family, the one you are raising, the one you are going to raise…”.* (Carlitos)

*”Being a woman is something that God has given us, so beautiful, at least for me… because being a woman I have been able to have my wonderful children that I love…”.* (Lila)

As it can be observed in the testimonies, ideas and concepts tinged with traditional stereotyped roles predominate, considering men as synonymous with physical strength and women as synonymous with sensitivity and reproductive role. Regarding sexuality, they consider that in order to be sexually attractive, women must have physical and aesthetic traits, while men must have traits of responsibility and respect:

*“Ah… let her be pretty, beautiful…”.* (Carlitos)

*”Uhm… Even if he has an important position, what prevails for me is his personality, that he is humble, simple and… even if he is not very handsome, for me, that is enough”.* (Evelyn).

Finally, it has been found that people with disabilities are frequently discriminated against, due to the denial of equality, leaving them “outside” based on the notion of normality/abnormality, as shown in the following testimony:

*“Yes, many times I have felt stigmatized, discriminated against… it hurt at the time… to make myself visible, it cost me a lot to be where I am, discrimination as a person with a disability and discrimination as a woman”. Gladys*.

Regarding the *Affective Bonding holon*, the participants explain that respect, humility, and communication are important in personal relationships, and during falling in love they are linked to affection; where love means making sacrifices for the happiness of the partner and that their presence alone “fills their world”. Likewise, they have experienced some feelings that became painful after a breakup.

They point out that behaviors for the construction and maintenance of interpersonal relationships are based on socially accepted norms:

*“Knowing how to listen, to know each other, uh… and to understand their weaknesses and strengths. Tolerance, dialogue, understanding, communication are fundamental”.* (Gladys)

*”Be kind, respectful, transparent.“* (Lila).

They consider that the valuation of friendship is based on presence and permanence, not necessarily physical:

*“Ah! I encapsulate it in one word: Loyalty!“.* (Lila)

*”Uhm…! I was very friendly before the accident, super friendly! I gave everything for my friends, the accident happened to me… not one of those friends went to visit me, not even to the hospital, not even to my house!* (Vita)

Regarding falling in love, they refer that it allows getting to know the partner, establishing affective bonds and courtship, searching for similar values, aimed at establishing commitments that may lead to marriage or cohabitation:

*“I think it is one more stage, of a sentimental bond, from a man to a woman, from woman to woman or man to man, it is one more step, if you could say, to take that step of the marital union.“* (Gladys).

*“For me, dating is the beginning of a relationship, of… trying to get to know each other, because later you live together or you get married. That’s when you get to know each other!”* (Jocesito).

They assume that marriage is the union of a couple that requires maturity, knowledge of the person, responsibility, communication, and support:

*“In marriage, to be a wife is to have a companion, to be a complement to the man, to be a person who can push you to work, together give love, form a home.“* (Gladys).

They emphasize that love is expressed through the search for the well-being of the couple:

*“Oh, love! Love I think… I can enclose it in one word: tenderness!“.* (Lila)

*”True love is sacrifice, if I get to love my partner or the person I chose, I have to sacrifice for her in many ways… in many ways…”* (Vita).

They consider that, during falling in love, there is a series of emotions and feelings, loss of sense, reason is clouded, everything is perfect, there is a strong desire to be together:

*“It’s feeling that… that there is someone in the world that makes you happy, that their very presence fills your world, fills all your emptiness, covers all your fears, more or less that.“* (Pequeña).

They also point out that there are feelings of disappointment, where the metaphorical expression “breaking the heart” refers to the disappointment, disillusionment and suffering due to the loss of a partner:

*“It is disillusionment, yes disillusionment. I have felt that my heart has been broken, by infidelity, by betrayal, I think that hurts everyone. I felt terrible, I wanted the earth to open up and… to lock me up. I felt a lot of pain…”* (Evelyn).

*“… It broke my soul when he said he couldn’t be with a person in a wheelchair.“* (Pequeña).

In the *eroticism holon*, male participants referred that intimacy is related to sexual satisfaction and is primarily coitocentric; for the female interviewees as a bond of peace, surrender, communication, and trust making their sexual life healthy because there is love.

It was observed that women reject masturbation and men consider pornography as a sexual stimulus. In general, the participants considered that amatory involves various forms of expression and explore their erogenous zones. However, after presenting the disability, several of the participants stated that their sex life ended, while others sought information to exercise it in their new condition. Men and women presented difficulties in sexual encounters due to lack of mobility and assured that both governments and health professionals are not interested in their sexual health, aspects that are corroborated by the following testimonies:

Women consider that intimacy refers to a space of peace and sublime purity, while for men it is related to sexual satisfaction:

*“For me intimacy is… it’s like a place, space, where two human beings kind of create a sacred circle, like a safe space, a space of infinite peace, very intimate, very pure very… very sublime…”* (Pequeña).

*“It’s… love, love with a woman, satisfaction, but… if you can’t, what are you going to do…”.* (Carlitos)

They stated that sexuality is considered the ultimate expression of love, communication, intimacy, and devotion:

*“Complementing a couple’s relationship, for me sexuality is the most important thing”* (Gladys).

*“I think it is the moment when two people commune, that is, live in a state of fullness, of… total surrender”.* (Pequeña)

They pointed out that sexual life is satisfying and fulfilling if trust, acceptance, tolerance, and communication of their preferences prevail:

*It is open, very trusting, that is, I can’t say: “Oh no, don’t look at me here! In terms of communication, we can tell each other what we like or what we don’t like…”.* (Evelyn)

*”Oh, the truth is that I do feel full! I feel very good… yes, yes… it makes me feel as if I am renewed, both inside and outside”.* (Pequeña)

It is thought that masturbation is exclusive to men because myths still prevail:

*“Once I tried to masturbate, but, I didn’t think it was nice because touching like that, it was like… who am I touching…, I don’t feel it’s pleasurable for me”.* (Gladys)

*”I think masturbation is a way to get sexual pleasure alone…I don’t know, in the male it’s different, a woman deflates through her period right…”.* (Evelyn)

They consider pornography as a means of individual stimulation, sexual encounter and as therapy in cases of erectile dysfunction.

*“Well, it can be a way to obtain stimulation in a sexual encounter, I think it’s excellent, we have used it on several occasions”.* (Gladys)

*”Pornography sometimes helps, as a psychologist I sometimes recommend it…, I am convinced that it helps in some situations.“* (Pequeña).

Some participants experienced discomfort when witnessing bondage and sadomasochism:

*“I admit that only once I saw…, but… I didn’t feel comfortable, because of the way they did it, that is, the oral sex and that, I saw that the woman had been handcuffed and was being beaten, I didn’t like it”!* (Evelyn)

In some participants, the centric coitus conception prevails:

*“It’s being more manly and having more satisfaction! Both for her and for me, that’s what there is through penetration then. … but now I can’t”.* (Carlitos)

Others have a more integral conception of sexuality, involving various forms of expression:

*“There is a lot of difference, for example, in sexual relations they think that it is penetration, but a sexual relation is… it is getting involved, it is caressing, kissing, playing, things like that, right”.* (Gladys)

Some claim that, from the moment they presented disability, they denied any possibility of sexual encounters:

*“A sexual relationship? It’s a passion, love, love as a couple, that is, during intercourse… but since my accident I have not had sexual relations at all, I think I have been affected, I guess I don’t know, because I am with a catheter, with a permanent bladder catheter…”.* (Lila)

Regarding erogenous zones, the interviewees agree that they experience a global pleasure that goes beyond the vagina or clitoris:

*“The breasts, the neck and he likes to have his penis touched, his neck.“* (Gladys).

*“I admit that more than the clitoris itself, what turns me on are the caresses of my nipples, my legs, my buttocks… I love those areas! And he knows it!“* (Pequeña).

They argue that, as sensitivity was lost or decreased in the genital area, it increased in other areas:

*“Uhm… a lot in the part of the armpits, abdomen, they touch me there… Their mother! Haha, if someone caresses my back I also like it”.* (Vita)

They associated sexual pleasure with love, respect, appreciation, rapport, and good stimulation of erogenous zones, generating a full sexual life:

*“Yes, I do achieve sexual pleasure, and that has happened to me because…it’s like, all the actions or forms of stimulation are good, so, they are pleasant…and that makes you reach that climax, I achieve orgasm, that happens to me! My sex life is healthy.* (Evelyn)

*”My sex life is nice, pleasurable, my sexuality was completely fine, I am multiorgasmic, I can experience two, three, four times.“* (Gladys).

They argue that difficulties in sexual encounters are attributed to lack of mobility:

*“I can’t move much because of my physical condition, I mean, I can’t move much, but he does, doesn’t he? I can’t support him… I would love to get on top of him, I can’t practice some things… "* (Pequeña).

*“My sex life before the accident, I was more active… definitely, I was more active, now because of, because of, because of the limb (leg) that I lost, I can’t do things that I used to do”.* (Josecito)

They affirm that sex education is a right; however, sexuality in disability is still a taboo:

*“I think, with no one, zero conversations on the topic of sexuality.“* (Carlitos).

*“Never, believe me, in the time that I have been working on the disability issue…, I proposed a project to the regional government to teach sexual orientation courses for people with disabilities. Believe me they didn’t, they didn’t accept…”* (Gladys).

Some report that they obtained the information from pornographic magazines, health professionals and friends:

*“A little bit, but apart from reading, uh… I like to interact sometimes with health professionals, friends who I hear them talk about in a sexual way…”* (Gladys).

*“From my family, they never, ever talked to me about sexuality. The information I’ve gotten, or who I’ve been able to talk to, is with friends. Sometimes it’s a little bit embarrassing”.* (Evelyn)

*”Ah! With my male friends, we touch on a lot of topics in the meetings we have for young people, we are a group of young single adults and so we have that freedom to be able to touch on those topics.“* (Vita).

Another aspect highlighted was the lack of sexual training of the health care team:

*“After I had my leg amputated, no doctor has talked to me about sexuality. Well I… I think they should have informed us because it’s not the same anymore, you can’t do the same thing…”* (Josecito).

Regarding the *Reproductivity holon*, the participants pointed out that motherhood and fatherhood is part of their life project, assuming the experience in a voluntary, responsible and planned manner, as an act of renunciation and dedication for the sake of their children, which motivates them to improve themselves.

They affirmed that pregnancy transcends the biological realm; it implies a voluntary decision, a meditated and planned event based on the couple’s desire.

*“Ah… actually, now I am not taking care of myself with any contraceptive method, I want to be a mother, we have already talked about it with my partner, now we have to wait…”* (Evelyn).

*“… He wanted to be a father and so did I, it was a mutual desire, both of us.“* (Pequeña).

They maintain that parenthood requires responsibility, willingness to provide children with everything necessary for their proper growth and development:

*“Father… although I am not a biological father, but I am a father… I have a daughter. Being a father has changed my life, I have someone to fight for, someone to live for. Before I didn’t, I took everything like that. She has definitely changed my life… she has made me more responsible”.* (Jocesito)

They emphasize that being a mother has great meaning and value, and implies commitment:

*“It’s the most beautiful experience. It’s a… it’s a letting go of thinking about yourself and feeling that you have to protect your baby. It’s the most sacred experience I would say, of total surrender, of giving up all your needs to meet the needs of this being that you have brought into the world.“* (Pequeña).

They point out that their families have violated their right to make reproductive decisions because of the myth that disability is hereditary:

*“…barriers that, for my situation are very enormous…I have gone through many very hard stages…for me life has been very complex…to my family I used to say Let me live! Let me make mistakes! They limit me because they are ashamed and want me to be at home! They were uncertain, because they thought that my children could inherit this disability”.* (Gladys)

## Discussion

In Peru, according to data from INEI, 1 out of every 10 people suffers from a disability, of which around 57% are women [10, 11]. Unlike other countries in the region, there are no health programs that respond to the sexual and reproductive rights of people with disabilities, guaranteeing their true integration into society and a full sexual life. This, together with the absence of comprehensive sexual education programs, results in the reinforcement of stereotypes and discrimination against people with disabilities.

In this regard, following Foucault’s philosophical proposal, the existence of a system of power that installs a “truth” to control, dominate the will and thought of individuals is evident; thus arising a process called “normalization“ [31] which generates discrimination and stigma [32] by cataloguing experiences as normal or pathological based only on social perceptions, a notion that does not apply to sexuality [33].

The results of the study show traditional conceptions regarding gender, giving men the role of protector and responsible for the family, and women the role of mother and wife. This coincides with the findings of Cruz [34] in relation to the fact that people with disabilities are assigned norms and values differentiated according to sex, which does not differ from that reported in other studies with a gender focus [35, 36].

It also showed that women with disabilities were more socially judged, perceiving double discrimination (as women and disabled). Other studies agree that women with disabilities are even denied the right to be wives, mothers and caregivers, due to social prejudices that consider them to be sick people who require care [37, 38].

Likewise, the study showed the need for people with disabilities to create strong emotional bonds that strengthen their interpersonal relationships as a key element for the enjoyment of sexuality, their ability to love, and to feel loved, valued and free to choose how to express and live fully.

It is difficult to talk about sexual encounters if one does not first learn to establish interpersonal relationships, which are established in social spaces. Hence the importance of including in health programs, spaces that strengthen these skills in people with disabilities, allowing better integration as social beings. This coincides with what has been stated by other authors regarding the importance of developing and promoting values, acceptance, support and respect for people with disabilities as tools to reduce stereotypes and avoid isolation and exclusion [39–42].

In terms of eroticism, the need for a satisfying and fulfilling sex life, regardless of disability, was evident. As other authors have observed [43], people with disabilities are often falsely described as “asexual”, without the same needs as people without disabilities [23, 44–46] It has been shown that women with spinal cord injury, where sexual capacity is significantly altered, do not necessarily present sexual dysfunctions, and can have an active and pleasant sexual life, as long as they receive information, education and early rehabilitation [47, 48].

With respect to sexual encounters, although some of them showed increased sensitivity in other areas of the body, men had a predominantly coitocentric conception, not recognizing other forms of sexual expression, generating feelings of annulment of their sexuality, especially in those who have lost sensitivity in the genital area. As for women, a more global conception predominated, which allows them greater well-being and satisfaction. These results coincide with those reported by other authors regarding the predominant sexual behavior, which reduces sexual encounters to genitality and, to a lesser extent, behaviors that consider love-making as an art that each couple creates with their own rules of the game, including all possible forms that are satisfactory to them [49], since every human being is ready to experience pleasure, which transcends the limitations associated with disability [50–55].

Although masturbation is part of the exercise of their sexuality and a practice that promotes sexual autonomy and self-knowledge, it is also a way of promoting their sexuality [44, 46] In this study, two currents were identified; while some said they practiced it naturally, others considered it harmful and sinful. As for pornography, two positions were also described; while for some it is considered a stimulus and even therapeutic for the good exercise of sexuality, for others it is uncomfortable and they reject it. Several authors have shown the influence of religion and cultural patterns in the experience of sexuality, exerting a negative social pressure on it [56, 57] and how sex education can change these patterns [58, 59].

These results reflect existing shortcomings in terms of scientific sex education by specialized professionals, as well as the influence of religion, society and Peruvian culture. In terms of reproductivity, the results showed the need to exercise maternity and paternity as a right of free choice, which requires a high level of responsibility and commitment to be assumed as a couple, which in some cases is limited by the prejudices of the close environment. This is in agreement with what has been reported by different authors, who point out that women with disabilities are often considered far from the health ideals for exercising their reproductive role, given the incompatibility due to their need for care and the lack of understanding and support from their surroundings [23, 60–62] The results show the need for social support programs, both economic and in healthcare, to guarantee the legitimate right to maternity and paternity of people with disabilities, since the existing ones in Peru do not assume this responsibility.

Regarding the experience of their sexuality, diversity is evident. While some experience it without fear; others have annulled their sexual encounters, because in addition to physical limitations, they experience psychosocial limitations such as pain, functional alterations, depression, low self-esteem, discrimination, exclusion, stigmas and socio-environmental barriers, among others [63, 64].

The perceptions that are presented about sexuality, sexual function and fertility potential are different, therefore, it is necessary to better understand all the changes after an injury. Patients and personnel involved in care must work together to improve the quality of sexual life [65].

Despite the fact that Peru has legislation that recognizes the right to sexual and reproductive health, there is no legislation that recognizes the right to sexual and reproductive health for this population group [15]. To date, no specific sexual and reproductive health programs have been developed, nor specialized training programs for professionals in charge of their care. This reflects the situation in the country and the region, where the sexuality of people with disabilities continues to be complex and ambiguous [6] A similar situation is evident in other countries, where the training of professionals in sexual health for people with disabilities is minimal [63, 66].

There are large gaps between sexual rehabilitation and support needs, considering that the acceptance of sexuality in these people is essential for a rapid integration into society and improve their quality of life [64].

There are many initiatives, health programs, advisory services, etc., implemented in countries such as Mexico [67] Argentina [68], Chile [69], Colombia [70], Costa Rica [71], among others [72], which are focused on improving the quality of life of people with disabilities. These efforts are examples of state strategies and policies that can be extrapolated to the Peruvian reality, given the need for trained professionals within the national health network to provide counseling or sex therapy to people with disabilities and their partners, as well as the importance of sex education, which is relevant due to the high percentage of the population with disabilities at the national level.

It is necessary to mention some limitations of the study that were primarily methodological: First, in the process of searching for potential participants, some were reluctant to participate because they considered it a taboo subject. Second, at the theoretical level, the difficulty was to identify similar studies, which complicated the development of the discussion of the findings. Finally, the mobility difficulties of the participants hindered the initial contact, so adjustments were made to protect their integrity and guarantee their comfort.

## Conclusion

Diversity in sexual experience was found. Some participants experience it without fear, others cancel their sexual encounters because, in addition to physical limitations, they experience psychosocial limitations such as: pain, depression, low self-esteem, discrimination, rejection, stigma, taboo, socio-environmental barriers, among others. Women present double discrimination, by gender and physical condition. There are no trained professionals for early care of sexuality in people with disabilities.

A review of public policies is suggested to make them more effective, since limitations in sex education, lack of sexological counseling and serious deficiencies in access to sexual and reproductive health care for people with motor disabilities continue to be found. On a practical level, it is recommended to review the role of the university in the training of professionals, especially in the health sciences, who have the capacity to respond to the needs of these people, with special emphasis on women’s sexual and reproductive health. On a theoretical level, researchers are urged to continue with these studies to strengthen them to include other types of disabilities and sexual orientations.

## Data Availability

The data sets used and/or analyzed during the current study are available from the corresponding author upon reasonable request.
